# Rational discovery of new capsaicin analogues as TRPV1 activators

**DOI:** 10.1186/1471-2105-16-S8-A8

**Published:** 2015-04-30

**Authors:** Javier Cáceres, Romina Sepúlveda, Camila Navas, Ramón Latorre, Fernando González-Nilo

**Affiliations:** 1 Center for Bioinformatics and Integrative Biology (CBIB), Andres Bello National University, Santiago, Chile; 2 Centro Interdisciplinario de Neurociencia de Valparaíso (CINV), Valparaíso, Chile

## Background

The ability to interpret environmental signals is a fundamental feature to ensure the integrity and survival of living organisms. The nociceptors are a group of sensory terminals that can detect a variety of noxious stimuli such as thermal or chemical stimuli, and are related with the generation of the pain signal and inflammation. These sensors have the ability to respond to capsaicin, the pungent agent of chilli peppers, which allowed the identification of TRPV1, a polymodal non-selective cation channel, tightly related with the generation of acute and neurogenic pain. TRPV1 is a member of the Transient Potential Receptor (TRP) family. It can be activated by noxious temperature (>42°C), low pH and ligands like capsaicin and resiniferatoxin. Even when the physiology of the channel has been characterized in some detail, the structural events that the channel undergoes during gating are still unknown. Recently the TRPV1 structure was resolved by electron cryomicroscopy in a fully closed conformation and in complex with activators such as capsaicin, allowing us to get insight of the distinct conformations of TRPV1. Consequently, the understanding of the structural background of the channel gating in a ligand-dependent manner provides exciting opportunities for pharmacological intervention.

## Results

The aim of this research is to identify novel activators of TRPV1 in a rational framework, taking the characterization of capsaicin as a model for structure-ligand interaction and to unravel the structure-function relationship involved in channel gating via molecular dynamics (MD) simulations. We have analyzed the vanilloid binding site within the third and fourth transmembrane segments, revealing a hydrophobic pocket with a marked presence of polar residues on the intracellular edge of the membrane. Furthermore, our MD assays has been shown that the capsaicin-dependent activation of the channel involves a shift in the curvature of the 'S6-TRP domain' segment disrupting the pore domain, presumably providing the first step for the ligand-dependent channel gating and thus revealing the great relevance of the vanilloid binding site to the channel opening. Molecular docking assays indicates the influence of the polar residues in the orientation of capsaicin in the binding pocket showing the vanilloid ring facing the polar residues and the aliphatic ramification pointing to the center of the membrane. These findings will allow us to perform a rational search strategy of novel TRPV1 activators, testing a database of 112.935 molecules with a massive molecular docking strategy. As a first approach we have selected a group of 10 molecules using the binding affinity and the hydrophobicity relative to capsaicin as selection criteria. Our goal is to contribute to the development of novel analgesic drugs and to increase the knowledge of the gating of this class of ion channels.

